# Supplementary material for: Single-Center Retrospective Analysis of Paraneoplastic Syndromes with Peripheral Nerve Damage
Source: Brain Sci. 2022 Dec 2;12(12):1656. doi: 10.3390/brainsci12121656 (PMC9775908; doi:10.3390/brainsci12121656)
Supplement: Supplementary file 1 [file brainsci-12-01656-s001.zip › brainsci-1922130-supplementary.pdf]

Table S1. Paraneoplastic neurological syndromes identified in our cohort, their associated neoplasms, onconeural antibodies and tumor markers, presenting mRS and duration of neurological symptoms at presentation.

Case #	Age sex	Tumor markers/Onconeural anti-body	Cancer	Syndrome	mRS	Time from symptom onset (months)
1	61 m	AFP(>1210), AFP-L3(>1210),AFP-L3/ AFP(>0.1),CA125(103.4),NSE(20.91),FER(454.2)	Liver cancer	MND	3	24
2	49 m	FER(488.9)	Lung cancer	CSMN	3	12
3	66 m	CEA(19.38),CA724(8.59),NSE(27.74)	Lung cancer	LEMS	3	5
4	47 f	Anti-Hu（+）	ND	SSN	3	24
5	69 m	CA125(39.74),CA153(48.91),CA724(7.55), NSE(26.52),FER(635.4)	Lung cancer	SSN	4	2
6	60 m	ND	Lung cancer	SSN	3	12
7	75 m	CY211(7.26),NSE(26.69)	Lung cancer	SSN	2	0.5
8	36 f	amphiphysin（+）	Breast cancer	CSMN	3	3
9	73 m	AFP(111.1), AFPL3(6.1),CA199(92.88), FER(609.10)	Liver cancer	SSN	1	1
10	60 m	CEA(58.14)	Cardia Cancer	SSN	3	12
11	73 m	FER(462.6)	Lung cancer	Dermatomyositis	3	0.7
12	68 m	ND	Lung cancer	SSN	4	2
13	64 m	ND	Transverse colon cancer	GBS	4	0.5
14	51 m	ND	Inguinal melanoma	SSN	4	11
15	59 m	FER(1079)	Lung cancer	SSN	5	3
16	65 m	(—)	Lung cancer	MND	4	12
17	60 m	CEA(10.11),FER(574.79)	Lung cancer	SSN	3	1
18	50 m	Anti-Hu（+），FER(713.8)	Lung cancer	CSMN	3	9
19	65 m	CA724(19.2),FER(505.5)	Kidney cancer	MND	4	36
20	57 m	NSE(16.92)	Lung cancer	MG	3	4
21	64 m	Anti-Hu（+），CA153(26.25),	Lung cancer	GBS	4	2
22	66 m	(—)	Esophageal cancer	SSN	2	2
23	67 m	(—)	Lung cancer?	CSMN	3	12
24	69 m	ND	Lang cancer	SSN	2	2
25	68 m	(—)	Lung cancer	CSMN	3	1

M = male, F = female, MND = motor neuron disease, FER=Ferritin, SSN=subacute sensory neuronopathy, GBS=Guillain-barre syndrome, CSMN=Chronic Sensorimotor neuropathies, ND=not done, LEMS=Lambert-Eaton myasthenic syndrome, MG=Myasthenia gravis, AFP=alpha-fetoprotein, AFP-L3=AFP- heterogeneity L3, CEA=carcinoembryonic antigen, CA125/153/724/199= carbohydrate antigens 125/153/724/199, CY21-1=cytokeratin 19 fragments, NSE =neuron specific enolase, tPSA=total prostate specific antigen, fPSA=free prostate specific antigen.

Table S2. The abnormalities of the recorded nerves.

	Nerve	DML	dCMAP/SNAP	MCV/SCV
Motor	Right-median. N	5/20	8/20	5/20
	Right-Ulnar. N	4/20	7/20	4/20
	Right-Peroneal. N	4/20	10/20	9/20
	Right-Tiabial. N	3/12	6/12	6/12
	Left-Peroneal. N	4/19	8/19	10/19

	Left-Tiabial. N	3/11	6/11	6/11
	Total	23/102, 22.55%	45/102, 44.12%	40/102, 39.22%
Sense	Right-Median. N		8/20	8/20
	Right-Ulnar. N		8/20	6/20
	Right-Peroneal. N		9/20	5/20
	Left-Peroneal. N		10/20	5/20
	Total		35/80, 43.75%	24/80, 30.00%

dCMAP: compound muscle action potential with distal; SNAP: sensory nerve action potential; MCV: conduction velocity of motor; SCV: conduction velocity of sensory; DML: distal motor latency.

Table S3. Comparison of abnormal rate of each parameter.

**Parameter 1**	**Parameter 2**	**X^2**	**P value**
Motor nerve abnormality 59/102（57.8%）	Sense nerve abnormality 39/80（48.8%）	1.49	0.22
amplitude 80/182（43.96%）	NCV 64/182（35.17%）	2.94	0.09
CMAP 45/102（44.12%）	DML 23/102（22.55%）	10.68	0.001
MCV 40/102（39.22%）	DML 23/102（22.55%）	6.64	0.01
CMAP 45/102（44.12%）	MCV 40/102（39.22%）	0.50	0.48
SNAP 35/80（43.75%）	SCV 24/80（30.00%）	3.25	0.07
Upper limbs nerve 39/80（48.75%）	lower limbs nerve 59/102（57.84%）	1.49	0.22
Upper limbs motor nerve 20/40（50.00%）	lower limbs motor nerve 38/62（61.29%）	1.26	0.26
Upper CMAP 5/40（37.50%）	Lower CMAP 30/62（48.39%）	1.17	0.28
Upper MCV 9/40（22.50%）	Lower MCV 31/62（50.00%）	7.71	0.005
Upper DML 9/40（22.50%）	Upper CMAP 15/40（37.50%）	2.14	0.14
Lower CMAP 30/62（48.39%）	Lower DML 14/62（22.50%）	9.02	0.003
Lower SNAP 19/40（47.50%）	Lower NCV 10/40（25.00%）	9.66	0.003

NCV: nerve conduction velocity; CMAP: compound muscle action potential; DML: distal motor latency; MCV: conduction velocity of motor; SNAP: sensory nerve action potential; SCV: conduction velocity of sensory;.

Table S4. Comparison between subacute sensory neuropathy and other syndromes.

	**Subacute Sensory Neuronopathy**	**Other Syndromes**	**X^2/ U**	***p* value**
Sensory nerve involvement	9/10(90%)	3/10(30%)	5.21	0.02
Complain of weakness	6/10(60%)	10/10(1000%)	30.00	0.03
Complain of paresthesia	8/10(80%)	2/10(20%)	7.20	0.01
Mean of Motor DML	0.80, 0.67	0.86, 0.11	32.00	0.19
Mean of dCMAP	1.28±0.74	0.95±0.72	1.01	0.33
Mean of SNAP	1.04±0.87	2.26±1.25	2.54	0.02
Motor nerve of lower dCMAP	1.17±0.84	0.93±0.77	0.68	0.51
Sensory nerve of lower SNAP	0.76±0.67	1.52±0.87	2.19	0.04
Sensory nerve of upper SNAP	1.32±1.19	3.01±1.92	2.36	0.03
Mean of NCV	0.94, 0.37	1.10, 0.17	20.00	0.02
Mean of Motor NCV	0.96, 0.46	1.08, 0.09	20.00	0.04
Mean of sensory NCV	0.92, 0.31	1.09, 0.08	28.00	0.11
Motor nerve of lower NCV	0.93, 0.51	1.01, 0.11	31.00	0.17
Sensory nerve of lower NCV	0.96, 0.29	1.06, 0.03	20.50	0.02
Sensory nerve of upper NCV	0.88, 0.28	1.10, 0.20	32.00	0.19

DML: distal motor latency; dCMAP: compound muscle action potential with distal; SNAP: sensory nerve action potential;; NCV: nerve conduction velocity;.
